# A cross-sectional analysis of orienting of visuospatial attention in child and adult carriers of the fragile X premutation

**DOI:** 10.1186/1866-1955-6-45

**Published:** 2014-12-11

**Authors:** Ling M Wong, Naomi J Goodrich-Hunsaker, Yingratana A McLennan, Flora Tassone, Susan M Rivera, Tony J Simon

**Affiliations:** Davis Medical Center, MIND Institute, University of California, Sacramento, CA 95817 USA; War Related Illness and Injury Study Center, Veterans Affairs Medical Center, Washington, DC 20422 USA; Department of Biochemistry and Molecular Medicine, University of California, Davis Medical Center, Sacramento, CA 95817 USA; Department of Psychology, University of California, Davis, CA 95616 USA; Center for Mind and Brain, University of California, Davis, CA 95616 USA; Department of Psychiatry and Behavioral Sciences, University of California, Davis Medical Center, Sacramento, CA 95817 USA

**Keywords:** Fragile X, *FMR1* gene, FXTAS, Endogenous, Exogenous, Cueing

## Abstract

**Background:**

Fragile X premutation carriers (fXPCs) have an expansion of 55–200 CGG repeats in the *FMR1* gene. Male fXPCs are at risk for developing a neurodegenerative motor disorder (fragile X-associated tremor/ataxia syndrome (FXTAS)) often accompanied by cognitive decline. Several broad domains are implicated as core systems of dysfunction in fXPCs, including perceptual processing of spatial information, orienting of attention to space, and inhibiting attention to irrelevant distractors. We tested whether orienting of spatial attention is impaired in fXPCs.

**Methods:**

Participants were fXPCs or healthy controls (HCs) asymptomatic for FXTAS. In experiment 1, they were male and female children and adults (aged 7–45 years). They oriented attention in response to volitional (endogenous) and reflexive (exogenous) cues. In experiment 2, the participants were men (aged 18–48 years). They oriented attention in an endogenous cueing task that manipulated the amount of information in the cue.

**Results:**

In women, fXPCs exhibited slower reaction times than HCs in both the endogenous and exogenous conditions. In men, fXPCs exhibited slower reaction times than HCs in the exogenous condition and in the challenging endogenous cueing task with probabilistic cues. In children, fXPCs did not differ from HCs.

**Conclusions:**

Because adult fXPCs were slower even when controlling for psychomotor speed, results support the interpretation that a core dysfunction in fXPCs is the allocation of spatial attention, while perceptual processing and attention orienting are intact. These findings indicate the importance of considering age and sex when interpreting and generalizing studies of fXPCs.

## Background

Fragile X syndrome (FXS) is caused by a trinucleotide repeat expansion (full mutation >200 CGG) in the *fragile X mental retardation 1* (FMR1) gene, which leads to gene silencing and subsequent reduction of *FMR1* mRNA and protein (FMRP). The fragile X premutation allele contains 55–200 CGG repeats, and it is estimated that 1 in 260–813 males and 113–259 females are carriers of the fragile X premutation allele (fXPCs) [1]. FXPCs are at elevated risk for developing a neurodegenerative disorder, fragile X-associated tremor/ataxia syndrome (FXTAS) [2]. The principle characteristics of this age-dependent disorder are intention tremor, ataxia, parkinsonism, and cognitive decline [3]. The *FMR1* gene is located on the X chromosome, and males lack a second X chromosome to compensate for the presence of the premutation allele, so men exhibit a higher prevalence of FXTAS than women.

### Motor impairment in FXTAS

The motor impairment characteristics of FXTAS are associated with the expanded CGG repeat length found in the premutation allele. In individuals with FXTAS, increased CGG repeat length was associated with earlier age of onset [4] and severity of motor symptoms [5]. Men with FXTAS were slower on a fine motor task [6] and exhibited increased intention tremor and postural sway using the CATSYS system [7]. This system was also sensitive to differences in performance between fXPCs with and without FXTAS [7] and between controls and fXPCs without FXTAS [8]. In the mouse model of the premutation, CGG knock-in (KI) mice exhibited motor impairments in a ladder rung [9] and skilled forelimb reaching task [10].

Motor impairments might be partially due to underlying problems processing space (visuospatial processing) or using visual representations to produce planned movements (visuomotor coordination). Visuospatial processing is primarily dependent on the magnocellular (M) layers of the lateral geniculate nucleus (LGN), which have relatively high FMRP levels [11]. The M layers provide input to cortical areas involved with motion perception, spatial vision, and visuomotor coordination, which contrasts with object recognition and color vision subserved by the parvocellular (P) layers of the LGN. Tasks biased towards demands on the M pathway, using psychophysical measures of sensitivity to biological and mechanical motion [12–14], were sensitive to a specific M pathway impairment in fXPCs. Additionally, M pathway function in healthy controls, who lack the premutation, relates to FMRP expression [15].

### Visuospatial processing impairment in fXPCs

Several studies report that fXPCs exhibit subtle yet significant visuospatial processing impairments [16–19]. Numerical processes, which build upon visuospatial processes, are also affected [20, 21]. Because these tasks are facilitated by areas such as the parietal cortex, these impairments suggest that parietal cortex structure or function might be atypical in fXPCs. Positron emission tomography (PET) in fXPCs observed hypometabolism in the parietal, temporal, and occipital cortices, which are critical for visuospatial processing [22]. Structural MRI studies indicate white matter involvement in these regions and in parietal projections of the cerebral peduncles [23, 24]. Complementing these findings in humans, CGG KI mice exhibit age-dependent decline in performance on a parietal cortex-dependent task [25], which might relate to the ability to overcome spatial interference [26].

### Other implicated functional domains

Orienting of visual attention is necessary for most visuospatial tasks and could be affected in fXPCs. For instance, when relatively large items must be counted, attention must be oriented to individual items in serial, leading to increases in reaction time to enumerate with an increasing number of items [27]. In this task, male fXPCs were slower than controls [18] and in female fXPCs, performance impairment related to CGG repeat length [16]. Importantly, stimuli were presented foveally, so no eye movements to shift attention were required.

In addition to the collected evidence for visuospatial processing impairment in fXPCs, other findings reveal visuomotor impairment on tasks such as block design [28, 29]. Meanwhile, several studies implicate attentional control impairment as a common feature in fXPCs with and without FXTAS [6, 30–33]. Thus, several broad domains are implicated as core systems of dysfunction in fXPCs: perceptual processing of spatial information, orienting of attention to space, inhibiting attention to irrelevant distractors, or using visual representations to plan and execute movements.

### The present study

The purpose of this study was to determine whether orienting of visual attention is dysfunctional in fXPCs asymptomatic for FXTAS. We assess orienting in terms of top-down, volitional attention, and in terms of bottom-up, reflexive attention. It is important to identify whether these abilities are intact, because they may affect performance in other tasks that are not designed to assess attention function, yet nonetheless require orienting of attention to perform the task. For example, performing a paper-and-pencil version of a Stroop task requires a continual orienting of attention to the next word, even if attention is captured when one realizes that one has made an error on the current word.

Although previous studies examined voluntary orienting ability when eye movements were not required, shifts in attention were small in magnitude. In real-world experience, shifts of attention occur over a larger visual angle range and are either volitional movements to endogenous cues (“What time is it? I should look at the clock”) or reflexive responses to exogenous cues (“Something just lit up over there. What is it?”). In experiment 1, we tested whether male and female adults and children who were fXPCs were able to effectively deploy volitional and reflexive shifts in attention. The lack of related literature did not justify any specific predictions regarding whether volitional or reflexive attention would be specifically affected in fXPCs. In the only relevant study, adult female fXPCs with impaired inhibitory control produced reflexive saccades of a typical latency, suggesting that reflexive orienting of attention was intact [34]. Thus, our goal was to describe the pattern in fXPCs.

Typically developing children younger than 8 years of age perform more poorly in volitional attention, but not reflexive attention, tasks than adults [35], which is thought to reflect immaturity of volitional attention at a young age. Thus, we expected that typically developing children in our sample, who were mostly older than eight, would not differ in performance relative to adults when controlling for psychomotor speed, although they would exhibit slower reaction times (RTs) [35]. Meanwhile, we predicted that the premutation allele would be associated with atypical development, such that children who were fXPCs would be impaired in the volitional attention task relative to control children.

Because males lack a second X chromosome, we expected that male fXPCs would be more affected than female fXPCs. We can find no other study that compares cognitive performance between children and adults who are fXPCs, so it is unclear whether we should expect a differential pattern of performance across age in fXPCs. However, because FXTAS is considered a neurodegenerative disorder, we predicted that in fXPCs, men would be more affected than boys. Therefore, adult males were selected as the subgroup for further testing in experiment 2. A subset of adult males from experiment 1 also completed experiment 2, which tested whether fXPCs were able to effectively use spatial information in cues to modulate the orienting of attention in space. Because a growing literature implicates visuospatial processing impairments in fXPCs, we predicted that fXPCs would exhibit impaired ability to use spatial information in cues. By using complementary cueing tasks, we determined whether different aspects of orienting ability were affected in fXPCs asymptomatic for FXTAS.

## Experiment 1

### Background

Experiment 1 examined the ability to volitionally and reflexively orient attention. We tested these abilities with a classic cueing paradigm using endogenous and exogenous cues, respectively. Exogenous orienting of attention requires bottom-up processing of visual information and develops at an early age, while endogenous orienting requires top-down control of attention, and develops at a later age [35]. We tested whether child or adult, male or female fXPCs were impaired relative to healthy controls (HCs) in either or both of these tasks.

### Methods

#### Participants

Participants were 194 male and female children (aged 7–14) and adults (aged 15–45), including 90 HCs (30 men, 31 women, 16 boys, 13 girls) and 104 fXPCs (25 men, 43 women, 21 boys, 15 girls). The participants were divided into adult and child age groups for two reasons: 1) to minimize confounds due to behavioral or cognitive changes during adolescence and 2) a distribution of ages during recruitment that resulted in relatively few participants aged 12–14 or 15–18 years. An additional five participants completed the task but were identified as outliers and excluded from all analyses (see outlier criteria in “Methods”); they were three men (2 HCs, 1 fXPC) and two women (1 HC, 1 fXPC). All the participants had normal, or corrected to normal, vision.

The participants were recruited through the Neuro Therapeutics Research Institute (NTRI) at the Medical Investigation of Neurodevelopmental Disorders (MIND) Institute at the University of California, Davis Medical Center and from the community through recruitment advertisements. FXPCs were recruited from known FXS pedigrees, and HCs were recruited from pedigrees or the community. Exclusion criteria were acute medical condition such as renal, liver, or cardiac or other disease that may be associated with brain atrophy or dysfunction; current or past history of major DSM-IV Axis I psychiatric disorder; history of head trauma, toxic encephalopathy, encephalitis, or bacterial meningitis; history of alcoholism or drug problem; and use of current medication that affect cerebral blood flow (e.g., beta blockers). This study was approved by the Institutional Review Board and conformed to institutional and federal guidelines for the protection of human participants. Written informed consent was obtained before participation from all the participants.

#### Procedure

The study visit involved administration of neuropsychological tests, a blood draw, and battery of cognitive tests. All fXPCs were evaluated by a physician and determined to be asymptomatic for FXTAS.

*Molecular assays.* Molecular data were *FMR1* CGG repeat length and mRNA expression level. Genomic DNA was isolated from peripheral blood leukocytes using standard methods (Puregene Kit, Gentra Inc., Valencia, CA, USA). Repeat length was determined using Southern blot analysis and PCR amplification of genomic DNA as described previously [36]. All quantifications of *FMR1* mRNA were performed using a 7900 Sequence detector (PE Applied Biosystems, Foster City, CA, USA). Due to difficulties in blood collection or processing, molecular data were not available from all the participants.

*Full-scale IQ (FSIQ).* FSIQ was measured using either the Wechsler Adult Intelligence Scale, third edition (WAIS-III) [37], the Wechsler Abbreviated Scale of Intelligence (WASI) [38], or the WISC-IV [39]. Due to time constraints during testing, FSIQ data were not available from all the participants.

*Simple reaction time (SRT) task.* The participants completed a manual simple reaction time task. Data from this task were used in experiments 1 and 2 to control for psychomotor speed. The task parameters were described previously [18]. Briefly, the participants responded as quickly as possible to the appearance of a stimulus by pressing a button. The stimulus appeared at randomized stimulus-onset asynchrony (SOA) so that its occurrence was unpredictable. Trials with anticipatory responses and outlier RTs were excluded from analysis. The outcome measure was median RT.

**Figure 1:**
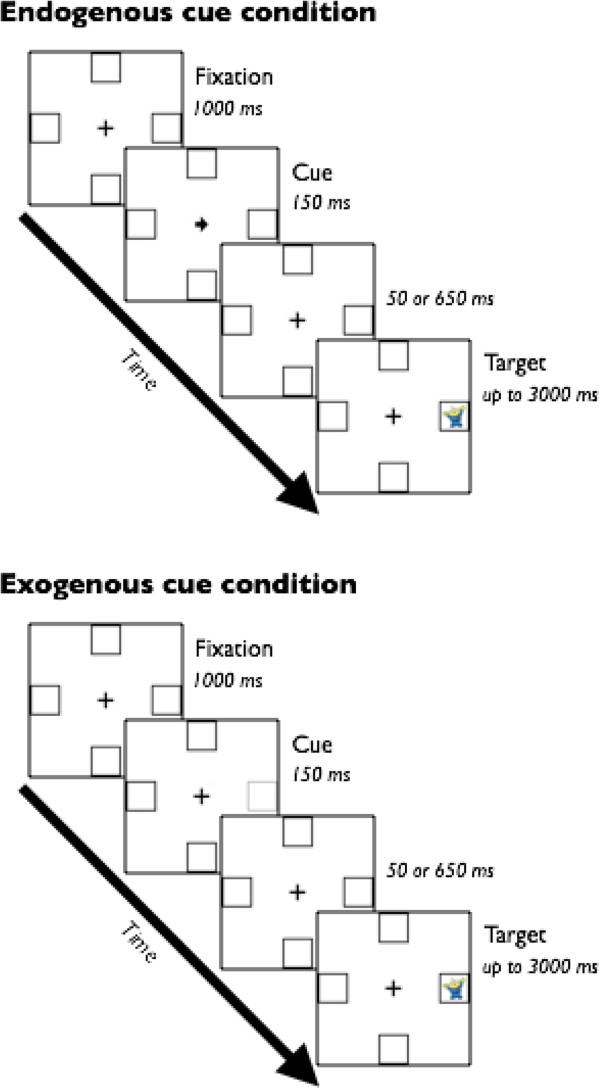
**Endogenous/exogenous cueing task design.** In invalid trials, the target appeared in the location opposite the cued location. In the endogenous cue condition, cues were valid in 80% of trials and invalid in 20% of trials. In the exogenous cue condition, cues were valid in 50% of trials.

*Endogenous/exogenous cueing task.* The task design and setup was identical to that used previously in our lab (Figure 1) [40], which was adapted from a previous design [41]. Briefly, the participants were seated 60 cm from the computer monitor, and they fixated on a cross in the center of the screen (0.95° × 0.95° visual angle (VA)) for 1,000 ms. A cue (150 ms) was used to orient attention to one of four locations. The locations were boxes that remained on screen throughout the experiment (1.91° in diameter, each located 3.82° VA from the center of the fixation cross). Targets appeared either in the cued location (valid cue) or the location directly opposite the cued location (invalid cue). Targets remained on screen until a response was made, up to 3,000 ms. The participants were instructed to keep their eyes on the cross and to, as quickly as possible, press the button corresponding to the spatial location of the target. After demonstrating proficiency during 10 practice trials, the participants performed a total of 160 experimental trials.

There were two cue conditions: endogenous and exogenous. Each cue condition consisted of 80 trials presented across two blocks. The order in which the two conditions were presented was randomized across participants. In the endogenous cue condition, the cue was a centrally presented arrow pointing toward one of the peripheral boxes. Cues were valid on 80% of the trials and invalid on 20% of the trials and were randomized across the experiment. In the exogenous cue condition, the cue was a luminance change in one of the peripheral boxes. This cue appeared with 50% invalid probability to avoid creating expectancy effects that could result in endogenous cueing. The arrow is considered an endogenous cue because an arrow does not inherently have meaning. We must learn that the arrow implies a rule (i.e., to look to where the arrow is pointing), and therefore, when we see an arrow, we must voluntarily shift our attention to that location. In contrast, a luminance change is considered an exogenous cue, because it is a perceptual stimulus that automatically grabs our attention, so we involuntarily shift our attention to that location.

#### Statistical analyses

Trials with anticipatory responses ( <150 ms) were excluded. Task RT values were divided by median SRT values to account for differences in psychomotor speed. All subsequent RT analyses presented in this study were performed on these normalized RT (NRT) values.

Within each cue condition, for each SOA and cue validity, trials whose RT was above or below the median by greater than three times the interquartile range (IQR) were identified as outliers and excluded. Eight group categories of the participants were identified, one for each age (adult or child), sex (male or female), and diagnosis group (HC or fXPC). Then, within each of the eight groups, for each cue condition, SOA, and cue validity, any participant whose RT was above or below the median by greater than three times the IQR was identified as an outlier and excluded.

Student’s *t*-tests were used for between-group comparisons of number of outlier trials and error rates. For each cue condition, a group (HC vs. fXPC) × sex (male vs. female) × age (child vs. adult) × validity ANOVA was performed on RT and NRT. Cue cost represents the cost of reorienting attention and was calculated as NRT to invalid trials minus NRT to valid trials. For each cue condition, a group × sex × age ANOVA was performed on cue cost. Significant interactions were further explored using Tukey’s HSD. Within-group comparisons in fXPCs were used to examine the effect of CGG and mRNA on NRT to valid cues.

### Results

#### Study sample

The participant characteristics are shown in Table 1. A total of 194 participants, aged 7.6–44.7 years, were included in analyses. The study sample was composed of 55 men (30 HCs, 25 fXPCs), 74 women (31 HCs, 43 fXPCs), 37 boys (16 HCs, 21 fXPCs), and 28 girls (13 HCs, 15 fXPCs). There was no difference in age between HCs and fXPCs in either male group (both *p* > 0.59), but both female groups of fXPCs were older than their control groups (both *p* < 0.01). To account for this, age was included in all models.

**Table 1:** **Experiment 1 participant characteristics**

		HC			fXPC				
		***N***	Range	Mean (SD)	***N***	Range	Mean (SD)	***t***	***p***
Men	Age (years)	30	18.9–40.7	30.8 (6.3)	25	18.0–44.7	30.1 (6.9)	0.55	0.59
	CGG	27	20–44	30.0 (4.6)	25	55–156	96.1 (25.3)	-12.9	<0.001
	mRNA	25	0.97–1.77	1.40 (0.23)	24	1.85–4.89	2.76 (0.77)	-8.24	<0.001
	FSIQ	25	85–148	118 (15)	24	79–143	114 (15)	0.91	0.37
Women	Age (years)	31	20.5–40.8	30.6 (6.5)	43	15.4–42.8	33.3 (5.9)	-2.59	0.01*
	CGG	29	28–47	31.2 (3.7)	43	67–178	95.6 (19.9)	-20.69	<0.001
	mRNA	28	0.9–1.98	1.41 (0.27)	43	1.55–4.62	2.39 (0.59)	-9.42	<0.001
	FSIQ	23	89–136	112 (13)	37	97–148	117 (13)	-1.31	0.20
Boys	Age (years)	16	7.6–12.8	10.5 (1.8)	21	8.4–13.1	10.4 (1.4)	0.10	0.92
	CGG	15	20–37	29.1 (4.7)	20	55–157	87.1 (30.7)	-8.31	<0.001
	mRNA	15	1.06–1.8	1.37 (0.24)	19	1.6–5.05	2.49 (0.88)	-5.31	<0.001
	FSIQ	12	101–139	120 (11)	11	73–113	97 (12)	4.72	<0.001
Girls	Age (years)	13	8.0–12.4	10.0 (1.6)	15	7.8–14.3	11.1 (1.9)	-2.40	0.02*
	CGG	10	25–34	30.5 (2.6)	15	57–147	98.1 (32.3)	-8.06	<0.001
	mRNA	7	1.02–1.8	1.39 (0.25)	14	1.28–5.19	2.61 (1.16)	-3.75	0.002**
	FSIQ	12	90–129	110 (12)	14	75–139	109 (19)	0.17	0.87

^*^ *p* < 0.05; **p < 0.01.

CGG data were missing from three men, two women, one boy, and three girls in the HC group (10%, 6%, 6%, and 23% of each group, respectively) and only one boy in the fXPC group (5% of boys). He was confirmed via pedigree analysis to be a fXPC. *FMR1* mRNA data were missing from five men, three women, one boy, and five girls in the HC group (17%, 10%, 6%, and 38% of each group, respectively) and one man, zero women, two boys, and one girl in the fXPC group (4%, 0%, 10%, and 7%, respectively). FSIQ data were missing from five men, eight women, four boys, and one girl in the HC group (17%, 26%, 25%, and 8%, respectively) and one man, six women, ten boys, and one girl in the fXPC group (4%, 14%, 48%, and 7%, respectively). Although the percentage of missing FSIQ data is relatively high for the adult female fXPC group, many women (*n* = 37) did have FSIQ data for analysis.

FSIQ did not differ between HCs and fXPCs for any subgroup except for boys, in which fXPCs had lower FSIQ scores than HCs (*t* = 4.72, *p* < 0.001). To examine whether these differences impacted behavioral performance, we tested correlations between FSIQ and NRT in the endogenous and exogenous cue conditions. Lower FSIQ was associated with slower NRT in the exogenous (*r* =-0.43, *p* = 0.04) but not endogenous (*r* =-0.32, *p* = 0.14) condition.

#### Behavioral performance

**Figure 2:**
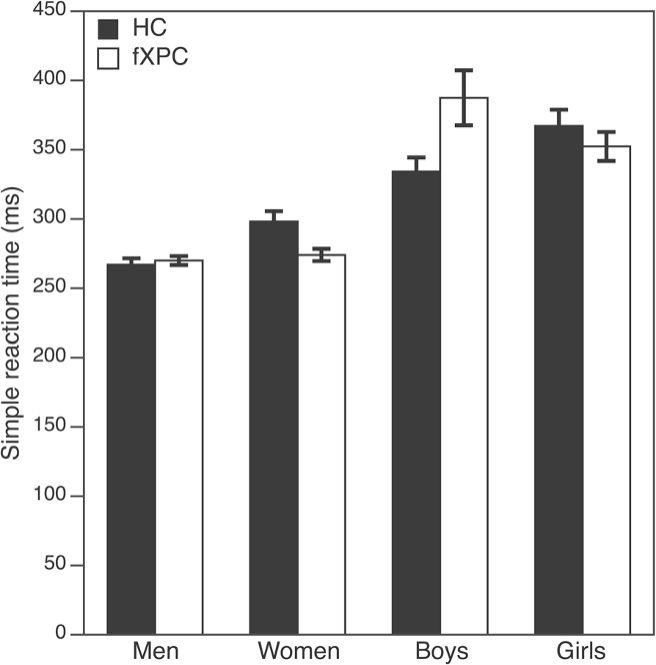
**Simple reaction time in the endogenous/exogenous cueing task.** There were no differences between HCs and fXPCs in men, women, boys, or girls (all *p* > 0.16). Error bars represent standard error of the mean (SEM).

*SRT.* Figure 2 shows SRT performance across groups. The median ( ± SD) SRT did not differ between groups of men (HCs: 267 ± 51 ms; fXPCs: 270 ± 32 ms; *p* = 0.79), women (HCs: 298 ± 82 ms; fXPCs: 274 ± 58 ms; *p* = 0.16), boys (HCs: 334 ± 82 ms; fXPCs: 387 ± 182 ms; *p* = 0.24), or girls (HCs: 367 ± 84 ms; fXPCs: 352 ± 81 ms; *p* = 0.64).

#### Endogenous/exogenous cueing task

*Outliers.* The number of trials identified as outliers and excluded from analysis was low. In the endogenous cue condition, out of 80 experimental trials, the mean number of excluded trials did not differ (all *p* > 0.16) between groups for men (1.07 vs. 0.68 in HCs vs. fXPCs), women (1.71 vs. 1.09), boys (2.36 vs. 3.24), or girls (2.54 vs. 3.13). Similarly, in the exogenous cue condition, the number of excluded trials did not differ (all *p* > 0.16) between groups for men (0.93 vs. 0.84), women (0.77 vs. 1.05), boys (2.75 vs. 3.19), or girls (2.62 vs. 1.27).

*Error rate.* Low error rates indicated that the participants were able to understand and successfully complete the task. In the endogenous cue condition, mean error rates did not differ (all *p* > 0.07) between groups for men (0.01 vs. 0.01 for HCs vs. fXPCs), women (0.02 vs. 0.01), boys (0.05 vs. 0.03), or girls (0.02 vs. 0.05). Similarly, in the exogenous cue condition, error rates did not differ (all *p* > 0.32) between groups for men (0.01 vs. 0.01), boys (0.05 vs. 0.02), or girls (0.02 vs. 0.02). Error rate did differ between groups (*p* = 0.03) for women (0.01 vs. 0.00), but because error rate was so low, this is unlikely to affect the pattern of results.

**Figure 3:**
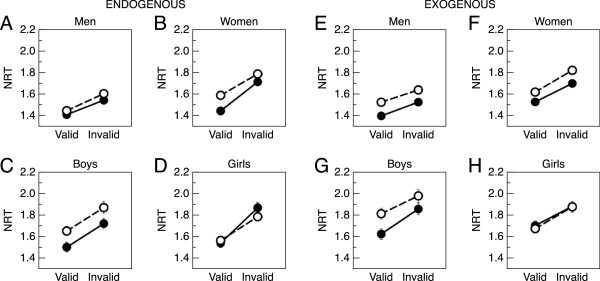
**Normalized reaction time in the endogenous/exogenous cueing task.** In both the endogenous **(A-D)** and exogenous **(E-H)** cue conditions, a significant main effect of validity was observed, such that NRTs were slower to invalid than valid cues (*p* < 0.001). There were also main effects of group, sex, and age (all *p* < 0.001), such that fXPCs, males, and children were slower than their comparison groups. The group × sex × age, group × sex, and group × age interactions were all significant (*p* < 0.001). Error bars represent SEM but are not visible when they are smaller than the data points.

*Normalized RT.* Results from the ANOVA on NRT are in Table 2. In both the endogenous and exogenous cue conditions (Figure 3), there was a main effect of validity such that NRTs were slower to invalid than valid cues ( *p*<0.001). There were also main effects of group, sex, and age (all *p* < 0.001), such that fXPCs, males, and children were slower than their comparison groups. The group × sex × age, group × sex, and group × age interactions were all significant (*p* < 0.001). The same pattern of results was observed using RT as the dependent variable instead of NRT (results not shown).

**Table 2:** **Experiment 1 group × sex × age × validity ANOVA on NRT**

	Endogenous		Exogenous	
	***F***	***p***	***F***	***p***
Group	39.61	<0.001	70.09	<0.001
Sex	235.69	<0.001	212.47	<0.001
Age	72.61	<0.001	204.27	<0.001
Validity	377.18	<0.001	311.88	<0.001
Group × sex	118.08	<0.001	139.68	<0.001
Group × age	49.54	<0.001	129.36	<0.001
Sex × age	16.56	<0.001	35.15	<0.001
Group × validity	12.30	<0.001	0.11	0.90
Sex × validity	12.91	<0.001	7.76	0.005**
Age × validity	5.41	0.02*	0.51	0.48
Group × sex × age	24.55	<0.001	24.78	<0.001
Group × sex × validity	2.28	0.10	1.85	0.16
Group × age × validity	0.93	0.39	3.35	0.04*
Sex × age × validity	0.15	0.70	1.30	0.25
Group × sex × age × validity	0.13	0.88	0.84	0.43

^*^ *p* < 0.05; **p < 0.01.

To explore the group × sex × age interaction further, a group × age × validity ANOVA was performed for each cue condition, for each sex. In both the endogenous and exogenous condition, a significant effect of group (*p* = 0.02 and *p* = 0.001, respectively) and age was observed in males (both *p* < 0.001). The effect of age was significant in the exogenous condition (*p* = 0.02) but not the endogenous condition in females (*p* = 0.30). As expected, the effect of validity (all *p* < 0.003) was significant for both sexes in both conditions. In sum, these results indicate that male fXPCs exhibited slower NRTs than male HCs, that male children exhibited slower NRTs than male adults, and that in the exogenous condition only, female children exhibited slower NRTs than female adults.

To examine our specific hypotheses, we then examined NRTs to valid cues within each cue condition. We examined whether typical children differed from typical adults and found no difference in the endogenous condition (adjusted *p* = 0.48), while children were significantly slower than adults in the exogenous condition (adjusted *p* = 0.02). We examined whether the premutation allele was associated with atypical development by comparing children who were fXPCs with HC children and found no difference in either condition (both adjusted *p* < 0.48). We examined whether men who were fXPCs were more affected than boys who were fXPCs and found no difference in the endogenous condition (adjusted *p* = 0.08), while boys were slower than men in the exogenous condition (adjusted *p* = 0.01).

**Figure 4:**
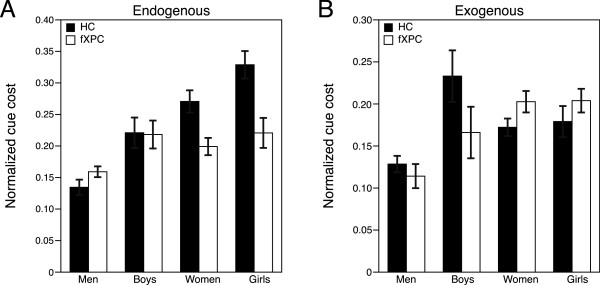
**Cue cost in the endogenous/exogenous cueing task.** **(A)** In the endogenous condition, the main effects of sex and age were significant, such that females exhibited greater cue cost relative to males (*p* = 0.006), and children exhibited greater cue cost relative to adults (*p* = 0.02). The group × sex interaction was significant, but follow-up analyses revealed that for both males and females, the groups did not differ in cue cost (adjusted *p* = 0.96 and *p* = 0.10, respectively). **(B)** In the exogenous condition, none of the effects or interactions were significant (all *p* > 0.13).

*Normalized cue cost.* ANOVA results for cue cost appear in Table 3. In the endogenous condition (Figure 4A), the main effects of sex and age were significant, such that females exhibited greater cue cost relative to males (*p* = 0.006), and children exhibited greater cue cost relative to adults (*p* = 0.02). The group × sex interaction was significant, but follow-up analyses revealed that for both males and females, the groups did not differ in cue cost (adjusted *p* = 0.96 and *p* = 0.10, respectively). In the exogenous condition (Figure 4B), none of the effects or interactions were significant (all *p* > 0.13). In sum, these results indicate that in the endogenous condition only, females exhibited greater difficulty reorienting attention than males, and children exhibited greater difficulty reorienting attention than adults.

**Table 3:** **Experiment 1 group × sex × age × validity ANOVA on normalized cue cost**

		Endogenous		Exogenous
	***F***	***p***	***F***	***p***
Group	1.35	0.25	0.04	0.85
Sex	7.64	0.006**	2.60	0.11
Age	5.26	0.02*	2.31	0.13
Group × sex	3.86	0.05*	1.62	0.21
Group × age	0.39	0.53	0.38	0.54
Sex × age	0.40	0.53	1.95	0.16
Group × sex × age	0.01	0.93	0.21	0.65

^*^ *p* < 0.05; **p < 0.01.

To examine our specific hypotheses, we performed follow-up analyses on cue cost within each cue condition. We examined whether typical children differed from typical adults and found no difference in either condition (both adjusted *p* > 0.19). We examined whether the premutation allele was associated with atypical development by comparing children who were fXPCs with HC children and found no difference in either condition (both adjusted *p* < 0.61). We examined whether men who were fXPCs were more affected than boys who were fXPCs and found no difference in either condition (both adjusted *p* > 0.56).

*Associations with molecular variables.* In fXPCs only, we tested for an association between performance (i.e., NRT or cost) and molecular variables (i.e., CGG repeat length or mRNA level). In both the endogenous and exogenous cue conditions, we found no significant associations between NRT or cost and CGG repeat length or mRNA level (all *p* > 0.05). This was observed whether we included all fXPCs; separately examined males and females; or separately examined men, women, boys, and girls.

### Discussion

The goal of experiment 1 was to examine the ability of fXPCs to volitionally (endogenous cue condition) and reflexively (exogenous cue condition) orient their attention in space. In this experiment, we compared groups of fXPCs of different ages and sexes (men, women, boys, and girls) to healthy controls. First, we expected that typically developing children in our sample would not differ in orienting ability relative to adults, although they would exhibit slower RTs [35]. Second, we predicted that the premutation allele would be associated with atypical development, such that children who were fXPCs would be impaired in the volitional attention task relative to control children. Third, because FXTAS is considered a neurodegenerative disorder and males lack a second X chromosome, we expected that male fXPCs would be more affected than female fXPCs, and that fXPCs who were men would be more affected than fXPCs who were boys. We will discuss each of these predictions in turn.

#### Development of endogenous and exogenous orienting

We expected that typically developing children in our sample would not differ in orienting ability relative to adults, although they would exhibit slower RTs. We reasoned that psychomotor speed and motor coordination are still developing in children, while volitional attention has mostly developed by age eight [35]. We observed an effect of age, such that in both cue conditions, boys exhibited slower NRTs than men, and in the exogenous condition only, girls exhibited slower NRTs than women. In support of our hypothesis, we observed that even when controlling for psychomotor speed, typically developing children exhibited slower NRTs than adults in the exogenous condition, but did not differ in cost. This indicates that although children took more time than adults to process the cue information to generate the appropriate response, they did not take relatively more time to reorient attention after an invalid cue.

#### Premutation allele across sex and age

We expected that male fXPCs would be more affected than female fXPCs, and that fXPCs who were men would be more affected than fXPCs who were boys. Our results supported our hypotheses, in that we observed interactions between group and sex, and main effects of each, in both cue conditions on NRT. First, we observed that in both cue conditions, male fXPCs were slower than male HCs. Although qualitatively it appeared that the slowing in boys relative to men was largely driven by slowing in young male fXPCs, the interaction of group with age on NRT was not significant. Second, we observed that the main effect of group on cue cost was not significant in either cue condition, indicating intact orienting ability in fXPCs. Third, although the group × sex interaction on cue cost was significant in the endogenous cue condition, follow-up analyses revealed that for both males and females, groups did not differ in cue cost.

We also predicted that the premutation allele would be associated with atypical development, such that children who were fXPCs would be impaired in the volitional attention task relative to control children. We found that children who were fXPCs did not differ from HC children either in NRT or cue cost in either cue condition. Lack of group differences in children might reflect a particular developmental trajectory in fXPCs, such that fXPCs truly do not differ from HCs as children, but that age-dependent differences emerge later in life. In short, we observed a sex-specific slowing in male fXPCs relative to HCs, intact orienting ability in fXPCs, and no evidence that fXPCs who are boys are particularly affected relative to HCs or fXPCs who are men. Notably, no group differences were observed in boys, even though there were IQ differences between groups and even though IQ correlated with performance in the exogenous condition. This indicates that although differences in overall levels of cognitive functioning had some impact on task performance, this impact was not great enough to produce group differences in performance.

#### Conclusions

In summary, because we controlled for psychomotor speed by dividing task RT by psychomotor RT, our results suggest that male fXPCs have slower cognitive (as compared to psychomotor) processing speed required to perceptually process stimuli, allocate attention, and generate a response. This might reflect that male fXPCs experience a greater cost when engaging their attentional processes, as opposed to engaging just a motor response. No interactions between group and validity on NRT were observed nor were effects of group on cue cost, indicating that fXPCs were able to orient attention appropriately to cued locations. These two findings, of greater attentional cost with intact orienting ability, indicate that fXPCs are specifically slower at perceptual processing (e.g., identifying the cue or target stimulus) or allocating attention resources (i.e., interpreting the cue and deciding where to shift attention). In the next experiment, we examined these possibilities in adult male fXPCs compared to HCs. We tested whether fXPCs were slower at perceptual processing by manipulating the saliency of the target and whether they were slower at allocating attention resources by manipulating the amount of information in the cue.

## Experiment 2

### Background

Experiment 2 examined the ability to use probabilistic cues to volitionally orient attention. We tested this ability by replicating a paradigm used by Hahn et al. [42], which was a modification of a classic Posner cueing task. This task requires the interpretation of endogenous centrally presented cues to orient attention, not just to one location in space but sometimes to two, three, or four locations in space. Because these cues have more or less information about where the upcoming target will appear, successful use of the cues to orient attention results in faster NRT when fewer locations are cued. Additionally, this task utilizes high- and low-saliency targets, which allows us to examine the effect of bottom-up perceptual processing. Specifically, if perceptual processing is intact, we should observe facilitated (faster) responses to high-saliency targets relative to low-saliency targets. Thus, compared to experiment 1, this task allows for a more detailed assessment of volitional orienting, while still allowing for an assessment of perceptual processing.

### Methods

#### Participants

Thirty-six male adults (17 HCs and 19 fXPCs) participated in experiment 2. All fXPCs were evaluated by a physician and determined to be asymptomatic for FXTAS. All the control participants completed the Tremor Disability Rating Scale [2]. Of 31 common actions, one control participant reported difficulty or disability on two actions (“using eyedrops” and “threading a needle”). Because this participant’s performance was not extreme, he was included in all analyses as a HC.

#### Procedure

Experiment 2 data were collected during the study visit described for experiment 1.

*ADHD assessment.* ADHD diagnoses are more prevalent, and symptoms are more elevated, in fXPCs relative to controls [43–46]. Adults with ADHD have been found to produce longer saccade latencies and increased anticipatory saccades [47]. Therefore, we measured ADHD status as a potential confound. ADHD status was measured using the 66-item Conners’ Adult ADHD Rating Scale (CAARS) [48]. The participants completed a self-report, and an observer-report was completed by a spouse, partner, family member, or close friend. Scores were adjusted according to established age and sex norms. Due to time constraints during testing and inability to collect observer reports during testing, ADHD data were not available from all the participants.

*Spatial attentional resource allocation task* (*SARAT*). The experiment was presented via E-Prime 2.0.8.90 (http://www.pstnet.com). The participants were seated 60 cm from the eye-tracking monitor in a chin rest to maintain head position. The participants were observed during task performance to ensure appropriate task performance. The task was begun only after the participant successfully completed practice trials to demonstrate understanding of task instructions.

**Figure 5:**
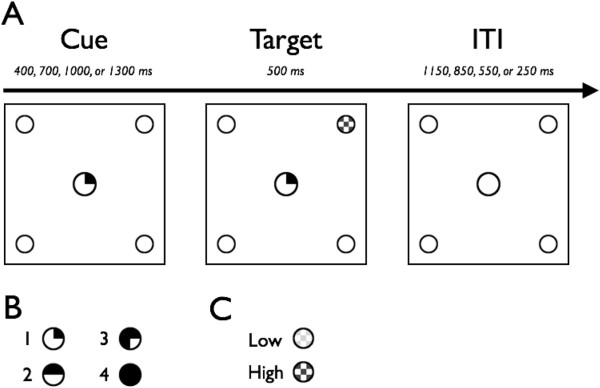
**Spatial attentional resource allocation task (SARAT) design.** **(A)** In each trial, a cue indicated that a target would appear in the cued location(s). Cues were valid in 80% of the trials and invalid in 20% of the trials. **(B)** Examples of each cue number. There were four cue conditions, in which 1, 2, 3, or all 4 locations were cued. **(C)** Examples of each target type. Targets were either low- or high-saliency.

This task (Figure 5) replicated parameters used by Hahn et al. [42]. The participants were instructed to fixate on a central circle containing a fixation cross, to use cues appearing within the circle to find the peripheral target, and to press a button as quickly as possible when the target was detected. Some participants noted that it was difficult not to look at the target once it was detected; they were instructed to return their gaze to the center once the target disappeared. The fixation circle and four circles in the corners of the screen remained on display throughout the experiment. The target was a 3 × 3 checkerboard pattern of one of two equally probable target intensities (high: grey squares were 80% grey; low: grey squares were 20% grey), appearing within one of the four corner circles. With the eyes in the center of the screen, the outer edges of the fixation circle were positioned at 1.3° – 1.5° VA, and the target circles were positioned at 10° – 12.5° VA.

The number of cued locations (1, 2, 3, or 4) was manipulated by darkening 1, 2, 3, or all 4 quadrants of the central circle. For example, a cue with the top half of the circle darkened indicated that the target would appear in one of the two top corners, with equal probability. Cues varied in duration (400, 700, 1,000, or 1,300 ms) and provided invalid information in 20% of trials with one, two, or three cued locations. The participants were informed that the cues were not always valid, but they should use the cues regardless. The target appeared for 500 ms, and the cue remained on screen until 500 ms after target offset. The inter-trial interval varied (250, 550, 850, or 1,150 ms) so that the duration of each trial was 2,700 ms.

In some trials, the cue was not followed by a target (“no-target trials”). In some trials, no cues or targets were presented, and the fixation cross remained on screen for the entire trial (“no-event trials”). These were interspersed throughout the task to jitter trial timing. All trials lasted for 2,700 ms. In each of 8 blocks, a total of 81 trials were presented: 32 valid trials, 16 no-target trials, 6 invalid target trials, and 27 no-event trials. All trial types were randomized within each block, and the order of trial presentation remained constant across the participants. The dependent measures were reaction time and accuracy in target detection.

#### Statistical analyses

Outlier trials were identified as trials with anticipatory responses or extreme RTs. Trials with RT less than or equal to 150 ms (anticipatory responses) were excluded. Then, for each cue validity, cue number, and target saliency, trials whose RT was above or below the median by greater than three times the IQR was identified as an outlier and excluded. Similarly, within each group, the participants whose RT was above or below the median by greater than three times the IQR was identified as an outlier and excluded.

As our laboratory has done previously, we used median SRT from a separate task to control for psychomotor speed. Therefore, the dependent measure of performance was RT in the SARAT task divided by SRT (“normalized RT” (NRT)). A group (HC vs. fXPC) × age × cue number (1–4) × saliency (low vs. high) ANCOVA was performed on NRT to valid cues. A second ANCOVA including the factor of validity (valid vs. invalid) was also performed for cue numbers 1–3. Finally, a group × age × cue number (1–3) × saliency ANCOVA was performed on normalized cue cost (calculated as NRT to invalid cues minus NRT to valid cues). Within-group comparisons in both groups were used to examine the effect of ADHD scores on NRT to valid cues. Within-group comparisons in fXPCs were used to examine the effect of CGG and mRNA on NRT to valid cues.

### Results

#### Study sample

The participant characteristics are shown in Table 4. Due to unexpected time constraints during testing, one HC did not complete the IQ assessment or blood draw.

**Table 4:** **Experiment 2 participant characteristics**

	HC			fXPC				
	***N***	Range	Mean (SD)	***N***	Range	Mean (SD)	***t***	***p***
Age (years)	17	24–40	31.4 (5.6)	19	22–48	31.6 (7.3)	-0.07	0.94
FSIQ	16	100–140	119 (9.8)	19	97–143	118 (12.8)	0.17	0.87
SRT (ms)	17	208–332	241 (24.7)	19	223–315	259 (27.9)	-0.82	0.42
CGG	16	20–44	30 (5.5)	17	55–146	101 (23.6)	-12.12	<0.001
mRNA	14	1.10–1.76	1.41 (0.2)	16	1.85–7.81	3.11 (1.39)	-4.84	<0.001

The mean age ( ± SD) was 31.4 ± 5.64 for HCs and 31.6 ± 7.25 for fXPCs, which did not differ significantly (*t* =-0.07, *p* = 0.94). CGG repeat length was available from 17 fXPCs, and mRNA level was available from 16 fXPCs (i.e., missing from two and three fXPCs, respectively). The two fXPCs lacking CGG repeat length data were confirmed via pedigree analysis to be fXPCs. The mean CGG repeat length was 29.56 ± 5.48 (range: 20–44) for HCs and 98.26 ± 23.54 (range: 55–146) for fXPCs, which differed significantly (*t* =-12.33, *p* < 0.001). One participant expressed two variants of the premutation allele (120 and 156), so the mean CGG value (138) was used for correlation testing. Because his performance was not extreme, he was included in all analyses. The mean mRNA value was 1.41 ± 0.21 (range: 1.10–1.76) for HCs and 3.11 ± 1.39 (range: 1.85–7.81) for fXPCs, which differed significantly (*t* =-4.84, *p* < 0.001).

FSIQ data were available from 16 HCs and 19 fXPCs (i.e., missing from one HC). Groups did not differ on FSIQ or SRT. Mean FSIQ was 119 ± 9.8 for HCs and 118 ± 12.8 for fXPCs and did not differ between groups (*t* = 0.17, *p* = 0.87). Mean SRT was 241 ± 24.7 for HCs and 259 ± 27.9 for fXPCs and did not differ between groups (*t* =-1.76, *p* = 0.09).

ADHD self-report data were available from 15 HCs and 18 fXPCs (i.e., missing from two HCs and one fXPC), and observer-report data were available from 13 HCs and 15 fXPCs (i.e., missing from four HCs and four fXPCs). None of the ADHD subscale scores differed between groups (all *p* > 0.61). No participants met ADHD criteria on both the self- and observer-report, though one HC and four fXPCs met ADHD criteria on the Total Symptoms subscale of the observer- and self-report, respectively.

#### Behavioral performance

*Outliers.* Although the number of trials identified as outliers were few (HCs: 4.14 ± 3.45, fXPCs: 2.74 ± 2.64), more outliers were identified for HCs than fXPCs (*t* = 5.62, *p* < 0.001). No participants were identified as outliers or excluded.

*Error rate.* Error rates were low for HCs (0.02 ± 0.02) and fXPCs (0.01 ± 0.01) and did not differ between groups (*t* = 0.87, *p* = 0.39).

**Figure 6:**
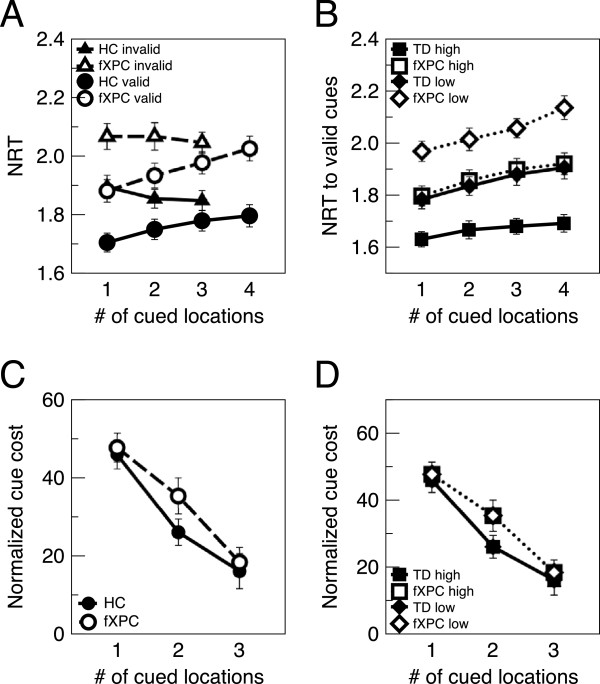
**SARAT performance.** **(A)** NRT to valid and invalid cues. Even after controlling for simple reaction time, fXPCs were slower than HCs (*p* < 0.001 in both the four-cue and three-cue ANCOVA). As expected, the effect of validity in the three-cue ANCOVA was significant (*p* = 0.02). Both groups showed a pattern of increased NRT with more validly cued locations, although the effect of cue number was not significant (all *p* > 0.17). **(B)** NRT to valid cues, for high- and low-saliency targets. As expected, the participants were slower to respond to low contrast targets than to high-contrast targets (*p* < 0.001 in both the four-cue and three-cue ANCOVA). FXPCs were as slow to respond to the high-contrast targets as HCs were to respond to the low-contrast targets (*t* =-0.97, *p* = 0.34). The interaction between group and saliency was not significant (both *p* > 0.76). The interaction between group and age was significant (both *p* < 0.001), such that NRT to valid cues increased with age in HCs (*r* = 0.60, *p* < 0.001) but decreased with age in fXPCs (*r* =-0.24, *p* = 0.003). **(C)** Cue cost for each number of cued locations. As expected, the effect of cue cost was significant, such that as the number of cued locations decreased, and the spatial predictability of the target increased, cue cost also increased (*p* = 0.004). **(D)** Cue cost for each number of cued locations, for high- and low-saliency targets. The effect of salience was significant, such that cue cost was greater for low-saliency than high-saliency targets (*p* = 0.009). Error bars represent SEM but are not visible when they are smaller than the data points.

*Normalized RT.* Table 5 shows the results of the ANCOVAs on NRT. The first ANCOVA included all four cue numbers as predictors, while the second ANCOVA included only cue numbers one to three. Because the four-cue cannot be invalid, the main effect or interactions with validity were not included in the first ANCOVA. Results from these ANCOVAs are shown in Figure 6A,B.

**Table 5:** **Experiment 2 group × age × validity × cue number × saliency on NRT**

	Endogenous		Exogenous	
	***F***	***p***	***F***	***p***
Group	20.44	<0.001	40.98	<0.001
Age	9.79	0.002**	9.02	0.003**
Validity	–	–	5.48	0.02*
Cue number	1.79	0.17	0.27	0.76
Saliency	20.41	<0.001	38.74	<0.001
Group × age	58.92	<0.001	89.76	<0.001
Group × validity	–	–	0.65	0.42
Group × cue number	0.07	0.93	0.16	0.86
Group × saliency	0.09	0.76	0.02	0.89

^*^ *p* < 0.05; **p < 0.01.

Figure 6A shows NRT for validly and invalidly cued trials. Even after controlling for simple reaction time, fXPCs were slower than HCs (*p* < 0.001 in both the four-cue and three-cue ANCOVA). As expected, the effect of validity in the three-cue ANCOVA was significant (*p* = 0.02). Both groups show a qualitative pattern of increased NRT with more validly cued locations, although the effect of cue number was not significant (both *p* > 0.17).

For display purposes, Figure 6B shows NRT for validly cued trials, though the analysis included invalidly cued trials as well. As expected, the participants were slower to respond to low-contrast targets than to high-contrast targets (*p* < 0.001 in both the four-cue and three-cue ANCOVA). FXPCs were as slow to respond to the high-contrast targets as HCs were to respond to the low-contrast targets (*t* =-0.97, *p* = 0.34). The interaction between group and saliency was not significant (both *p* > 0.76), suggesting that differences in perceptual processing time (facilitated responses for high-saliency relative to low-saliency targets) are not responsible for the group differences found in NRT. The interaction between group and age was significant (both *p* < 0.001), such that NRT to valid cues increased with age in HCs (*r* = 0.60, *p* < 0.001) but decreased with age in fXPCs (*r* =-0.24, *p* = 0.003). Thus, fXPCs were slower than HCs but showed an improvement in performance with age.

*Cue cost on normalized RT.* Table 6 and Figure 6C show the results of the ANCOVAs on normalized cue cost. As expected, the effect of cue cost was significant, such that as the number of cued locations decreased, and the spatial predictability of the target increased, cue cost also increased (*p* = 0.004). As seen in Figure 6D, the effect of salience was significant, such that cue cost was greater for low-saliency than high-saliency targets (*p* = 0.009).

**Table 6:** **Experiment 2 group × age × cue number × saliency on cue cost**

		One-cue to three-cue
	***F***	***p***
Group	0.15	0.70
Age	1.25	0.27
Cue number	5.82	0.004**
Saliency	7.01	0.009*
Group × age	0.55	0.46
Group × cue number	0.13	0.88
Age × cue number	1.54	0.22
Group × saliency	0.01	0.95
Age × saliency	1.34	0.25
Cue number × saliency	0.13	0.88
Group × age × cue number	1.09	0.34
Group × age × saliency	0.00	0.95
Group × cue number × saliency	0.51	0.60
Age × cue number × saliency	2.33	0.10
Group × age × cue number × saliency	0.23	0.79

^*^ *p* < 0.05; **p < 0.01.

#### Associations between task performance and other measures

*ADHD scores.* We tested for an association between performance and ADHD scores within each group. We found that in fXPCs, NRT to valid cues was negatively correlated with self-report of ADHD symptoms in the total score (*p* = 0.04), inattention score (*p* = 0.04), and index score (*p* = 0.03). In other words, lesser self-reported ADHD symptoms were associated with worse performance, suggesting that fXPCs may have difficulty assessing their attention problems. There were no significant associations between performance in fXPCs and observer-report or between performance in HCs and either self- or observer-report.

*Molecular variables.* In fXPCs only, we tested for an association between performance and molecular variables. We found that NRT to valid cues increased with increasing CGG repeat length (*r* = 0.49, *p* = 0.03), but not with increasing mRNA level (*r* = 0.05, *p* = 0.85).

### Discussion

FXTAS is a neurodegenerative disorder that predominantly affects males and is more likely to occur in older individuals. Thus, any signs of dysfunction related to cognitive decline associated with FXTAS are most likely to be observed in adult male fXPCs. In experiment 1, we found that male fXPCs were slower than male HCs in both volitional and reflexive attentions. Given this theoretical justification and experimental finding, experiment 2 was performed in adult male fXPCs. In the second experiment, we expected that factors other than cue validity, such as amount of information in the cue or salience of the target, would also impact group performance. Thus, the goal of experiment 2 was to examine the ability of fXPCs to use probabilistic cues to volitionally orient attention. We hypothesized that in this challenging volitional orienting task, fXPCs would exhibit impairment relative to HCs.

First, we found that fXPCs exhibited slower RTs than HCs, even when controlling for psychomotor speed. This suggests that male fXPCs experience greater cost when engaging attention, as opposed to engaging a motor response. Second, we found that fXPCs were able to use probabilistic cues to volitionally orient attention. This was observed as a pattern of faster RT when fewer locations were cued, which did not differ from HCs. Third, we found that fXPCs were not slower than HCs at reorienting attention when the cue was invalid. This was observed as a lack of group × validity interaction. Fourth, both groups were slower to respond to low-saliency targets than to high-saliency targets, but there was no group × saliency interaction on NRT, suggesting that perceptual processing was intact in fXPCs. Fifth, group interacted with age, such that NRT increased with age in HCs but decreased with age in fXPCs. Because RT performance across the life span typically follows an inverted U-shaped function, one possibility is that the typical RT improvements observed in young adulthood in HCs is delayed in fXPCs. Further testing of fXPCs in a younger age range is needed to assess this possibility. Finally, cue cost did not differ between groups. This indicates that when controlling for psychomotor speed, the ability to orient attention in response to cues was intact in fXPCs.

In summary, results from experiment 2 demonstrate that adult male fXPCs exhibited volitional attention impairments relative to HCs, as measured by slower RTs. Other aspects of performance, specifically perceptual processing and ability to use cue information to effectively deploy attention, were intact in fXPCs.

## Discussion

### Summary of findings

Several broad domains have been implicated as core systems of dysfunction in fXPCs: perceptual processing of spatial information, orienting of attention to space, inhibiting attention to irrelevant distractors, or using visual representations to plan and execute movements. However, studying these functions in isolation makes it difficult to identify the root cause of dysfunction, if there is one. For example, fXPCs might perform poorly on an executive function task only because spatial stimuli are used, and fXPCs take longer to perceptually process spatial information. We aimed to better specify the fXPC phenotype by determining whether orienting of visual attention is dysfunctional in fXPCs asymptomatic for FXTAS.

In experiment 1, we tested whether fXPCs were able to effectively deploy volitional and reflexive shifts in attention. We compared fXPCs who were men, women, boys, or girls to HCs. We observed a similar pattern of results whether or not we controlled for psychomotor speed. We found that: (1) typically developing children exhibited slower NRTs than adults in the exogenous condition but did not differ in cost; (2) children who were fXPCs did not differ from HC children either in NRT or cue cost in either cue condition; (3) male fXPCs were slower than male HCs, while female fXPCs did not differ from female HCs; and (4) boys who were fXPCs were not significantly more affected than men who were fXPCs.

In experiment 2, we tested whether men who were fXPCs exhibited intact endogenous cue performance when cues could orient attention to multiple locations and when targets were perceptually easy or difficult to detect. We found that: (1) fXPCs were slower than HCs, even after controlling for psychomotor speed; (2) fXPCs exhibited an intact ability to use probabilistic information within cues to orient attention; (3) fXPCs exhibited an intact ability to effectively reorient attention when the cue was invalid; (4) fXPCs exhibited intact perceptual processing of targets; and (5) NRT increased with age in HCs but decreased with age in fXPCs.

### Implications

Both these experiments indicated that male fXPCs produced slower responses than HCs on a range of attentional functions, even when controlling for psychomotor speed. However, orienting of attention, assessed as the validity effect, was intact in fXPCs. Together, these two results suggest that fXPCs exhibited a specific impairment in allocating spatial attention. This may be due to increased time required to interpret the information in the cue (i.e., “this cue means the target will appear over here”), which is distinct from simply viewing a non-informative stimulus (i.e., “press a button whenever something appears”). Because fXPCs, like HCs, exhibited faster RTs to more informative cues, we conclude that once a cue is processed, fXPCs are able to use that information to modulate their behavior.

Bottom-up processing was differentially assessed across the two experiments. In experiment 1, the bottom-up process was reflexive orienting or the ability to shift attention in response to exogenous cues. In experiment 2, the bottom-up process was perceptual processing or the ability to detect high-saliency targets faster than low-saliency targets. Interestingly, adult fXPCs were found to be impaired in the former, but not the latter. This differential finding suggests that ease of detection of external stimuli is intact in fXPCs, but that the ability to then shift attention to those stimuli is slower in fXPCs than HCs.

These results are consistent with the previous findings in fXPCs. The M pathway is involved in functions such as spatial vision, which is critical for orienting of spatial attention. Tasks biased towards demands on the M pathway, using psychophysical measures of sensitivity to biological and mechanical motion [12–14] were sensitive to a specific M pathway impairment in fXPCs. Additionally, M pathway function in healthy controls, who lack the premutation, relates to FMRP expression [15]. This relationship provides a potential biological basis for observed differences between groups. Of note, our finding that fXPCs exhibited increased cost in engaging spatial attention does not necessarily implicate M pathway involvement in fXPCs but rather is consistent with that model. Specifically, high- and low-saliency targets were not designed to be biased toward M pathway processing, and we did not observe group differences in perceptual processing of targets. Meanwhile, our stimuli were visual and spatial in nature, characteristics which are biased toward M pathway processing, and we observed group differences in RTs to these stimuli.

Our finding of a group effect in both adult males and females indicates that attention orienting is impaired in fXPCs both sexes. This indicates that, at least in this cognitive domain, the second, unaffected X chromosome in females is insufficient to exert protective effects. Because we did not observe volitional attention impairment in men who were fXPCs in experiment 1, but we did observe an impairment in experiment 2, we conclude that sufficiently challenging tasks are required to detect subtle cognitive impairment in fXPCs.

We found that children who were fXPCs performed similarly to HCs. This may be because the small sample size we were able to recruit was insufficient to detect subtle group differences. Alternatively, this may reflect that the maturation of attention systems is largely intact in fXPCs but follows an atypical trajectory in fXPCs. The group × age interaction in experiment 2 suggests the importance of considering potentially nonlinear effects of age, and whether the effects of the premutation are best modeled via neurodevelopmental or neurodegenerative processes.

### Limitations

Interpretations of the results of these experiments are limited in several ways. Our first limitation has to do with sample size. Not all men who completed experiment 1 also completed experiment 2. This is because we used our preliminary results from experiment 1 to design experiment 2. Thus, while we draw conclusions on results common to both experiments, we acknowledge that experiment 2 has a smaller sample size. Additionally, the groups of children have relatively small sample size. This was due to difficulty in identifying and recruiting children who were fXPCs. Because there are very few studies of children who are fXPCs, this is likely a common difficulty and must be addressed with more advanced recruiting techniques to produce larger sample sizes. Meanwhile, our inclusion of both children and adult fXPCs, and inclusion of age as a factor in our analyses, is a strength of our study.

Second, although we draw conclusions about the development of the ability to orient attention across age, this was a cross-sectional study. Longitudinal studies are needed to demonstrate whether our findings accurately represent the proposed developmental trajectory.

Third, we did not control for the use of overt or covert attention to complete the tasks. Although the participants were instructed to remain fixated on the center, we could not and did not exclude trials in which eye movements were made. This is a limitation of many behavioral studies of attention, and we do not know how our results might differ if we were to exclude trials with eye movements toward the target. Despite this limitation, tasks from both experiments 1 and 2 have been used in the previous studies and produced predictable results [40, 42]. Given the current literature, we feel there is insufficient evidence to predict that fXPCs would exhibit a specific impairment in either overt or covert attention.

Fourth, although the tasks used in experiments 1 and 2 test similar domains, they differ in several respects. While these differences made the second task more challenging and sensitive to group differences, they also limit the extent to which we can interpret and generalize results across experiments.

Finally, an impairment in some other domain might underlie our finding of impaired orienting of visual attention. For example, fXPCs might exhibit impairments in priming more generally, which is not specific to the spatial domain. One study found that the N400 repetition effect, an event-related brain potential which is thought to reflect semantic priming or other implicit memory processes, was reduced in size in adults with FXTAS [49]. However, the participants in our sample were asymptomatic for FXTAS, and we do not know if fXPCs asymptomatic for FXTAS would also exhibit a reduction in the N400 repetition effect. We can identify no other studies of priming in fXPCs, so further research is needed to clarify whether altered priming might explain our pattern of results. Future visual cueing studies using only centrally presented stimuli would allow for determination of whether spatial orienting is particularly affected in fXPCs.

## Conclusions

By using complementary cueing tasks, we determined whether different aspects of orienting ability were affected in fXPCs asymptomatic for FXTAS. We found that male fXPCs exhibited increased cost in engaging visual spatial attention relative to HCs, measured as longer RT even when controlling for psychomotor speed, and there were no significant differences between children and adults who were fXPCs. This was the case when either a volitional or reflexive shift of attention was required. We also observed that adult male fXPCs did not differ in their ability to use varying amounts of information in cues to differentially modulate their behavior. This pattern of longer RT but intact RT modulation suggests that a core dysfunction in fXPCs is the increased cost to allocate spatial attention to modulate behavior, while perceptual processing and attention orienting are intact. Because inhibitory control dysfunction is often reported in fXPCs, we suggest that to reduce the presence of potentially confounding factors, future tests of inhibitory control might benefit from minimal use of spatial information.
